# COVID-19 and Distance Learning: Effects on Georgia State University School of Public Health Students

**DOI:** 10.3389/fpubh.2020.576227

**Published:** 2020-09-25

**Authors:** Elizabeth Armstrong-Mensah, Kim Ramsey-White, Barbara Yankey, Shannon Self-Brown

**Affiliations:** School of Public Health, Georgia State University, Atlanta, GA, United States

**Keywords:** COVID-19, distance learning, Georgia State University School of Public Health, students, course transition, school closure

## Abstract

On March 11, 2020, the World Health organization declared COVID-19 a global pandemic. Following the speed with which COVID-19 spread to all parts of the world, and to contain the spread of the disease, most governments around the world, including the US, authorized unprecedented social containment measures to stem the tide. These measures among others required social distancing and the temporary physical closure of educational institutions. The Georgia State University School of Public Health, like all other institutions of higher learning, had to create distance-learning opportunities to enable students to complete the 2019–2020 academic year. The unplanned, rapid, and uncertain duration of the approach presented challenges at all academic levels. Not much information on best practices was available to guide such abrupt transitions to college education. The purpose of the study was to collect data on how the transition to distance learning impacted undergraduate and graduate students taking courses in public health at GSU. The goal was to identify student academic challenges and the unforeseen benefits of distance learning, and to use that information to inform practices that can be implemented during crises that impact university education.

## Introduction

On December 31, 2019, Chinese authorities reported to the World Health Organization (WHO), the presence of numerous cases of an unknown pneumonia-like disease that presented like flu in Wuhan City, Hubei Province in China (1). After virus isolation and analysis of the viral genome sequence from the lower respiratory tract samples of infected patients, a novel coronavirus named severe acute respiratory syndrome-related coronavirus 2 or SARS-CoV-2 was identified and subsequently named COVID-19 by the WHO (1). A month after its emergence, the WHO declared COVID-19 a global pandemic and a day later, the United States (US) declared the disease a public health emergency (2). By May 27, 2020, the WHO had confirmed 5,488 million cases of COVID-19 in over 180 countries, with about 1.634 million of those cases occurring in the US (3, 4).

With no successful vaccine or treatment available, and in an attempt to contain the spread of COVID-19, most governments around the world, including the US, authorized unprecedented social containment measures. These measures, among others, included social distancing and the temporary physical closure of educational institutions. Educational institutions had to adopt a digital approach to instruction and student learning, dramatically transitioning traditional in-person classroom instruction to predominantly distance learning where teaching is provided remotely on digital platforms. At present, there are over 300 college and university closures in the US, affecting millions of students (5). While distance learning is not a new approach to instruction and learning at Georgia State University's (GSU) School of Public Health (SPH), the unplanned, rapid, and uncertain duration of the approach, is presenting challenges and taking a toll on students at all academic levels. Not much information on best practices was available to guide such abrupt transitions to college education. The purpose of this study was to collect information on how the transition to distance learning impacted undergraduate and graduate students taking courses in public health at GSU. The goal was to identify student academic challenges and unforeseen benefits of distance learning, and to use that information to inform practices that can be implemented during future crises that impact university education.

## Distance Learning and GSU SPH Students

From time immemorial, faculty lecturing in a classroom setting, students listening, taking notes, asking questions, and getting those questions answered have been the backbone of traditional academic education (6). With advancements in communication technology such as the telephone, radio, television and most recently the internet, new methods of learning, including distance learning, have emerged (7). Through the internet, students can now obtain instruction and learn with ease at home by simply clicking a few buttons on the computer to listen live or asynchronously to a professor thousands of miles away, interact with the professor, and solve problems without having to physically be in a classroom (6). While a more expensive option for education in terms of set up, distance education has progressed in concept and practice from an “anywhere” to an “anytime” education delivery method (8).

Distance learning, also known by various names such as distance education, e-learning, mobile learning, or online learning, is a form of education where there is physical separation of teachers from students during the instruction and learning process (9). It is also an instructional practice that effectively utilizes a wide range of tools and technology to enrich the student learning experience (10) and to facilitate student-faculty and student-student communication (9). The minimum technological requirements for successful distance learning include the acquisition of hardware such as a computer, mobile device (cellular phones), or webcam, some form oflistening device, video conferencing applications such as WebEx or Zoom, Microsoft Windows or Apple operating systems, and a stable internet connection with a speed of about 56K (56,000) or greater (11).

The GSU SPH is a Council on Education in Public Health (CEPH) accredited school, located in the heart of downtown Atlanta, an area often referred to as the public health capital of the United States. Given the school's close proximity to leading national, state and local public health institutions, our school attracts many students interested in public health education. The GSU-SPH is one of the fastest growing schools of public health in the US and offers degree programs at the Bachelors, Masters and Doctoral levels. The program was established in 2006 and has grown to more than 700 students enrolled across all of the degree programs. As a school of public health with a diverse student body, where 67% are non-white and 10% of the alumni are Fulbright scholars, the academic and technological needs of the student body during the times of this pandemic are exceptionally broad. Despite the challenges of meeting the various needs of the students, the GSU-SPH worked diligently to ensure that access to and quality of instruction for all students was the highest priority during the turbulent spring 2020 semester.

Given the fact that the GSU SPH student body like other schools and colleges on campus comprises traditional and non-traditional students, a combination of classroom, fully online and hybrid courses are offered each semester. Although initially geared toward non-traditional GSU SPH adult learners such as full-time workers, and individuals who were unable to attend classroom lectures in person, distance learning has become an established part of the GSU SPH curriculum and an option for some traditional students as well. What is at issue with distance learning in the COVID-19 environment is the lack of options for students to determine whether they want to take online courses or not, the lack of access to free technology hardware, software and internet services on campus due to social distancing, a lack of motivation to learn; the new course workload, adapting to unfamiliar technology for first time online student users and uncertainty about the future among others (12).

Making distance learning work for all students is challenging. The best tools can be in place, but without equitable access by all students to the tools, adequate preparation time and training for faculty, and the adaption of existing curricula, or the development of brand-new course syllabi, it will be difficult to replicate the in-person learning experience, online. Consequently, some questions that have arisen with distance learning is whether it offers the same value as learning in a classroom, and whether it helps students to imbibe knowledge as they would if they were in a classroom? While these questions are relevant, there are additional significant issues that institutions of higher learning, have to consider, such as how to assist students without reliable internet access and/or technology to participate in digital learning. The disparity in access to technology and internet access is especially glaring among minority populations. While some universities have been able to provide digital equipment to students in need, some including GSU SPH have not been able to adequately provide this service and are concerned that the pandemic will widen the digital divide between students and thus, negatively impact their education.

## Materials and Methods

### Setting and Population

The GSU SPH has 769 traditional and non-traditional students of varied racial and socio-economic backgrounds enrolled in five different public health programs—the Bachelor of Science in Public Health, Graduate Certificate, Master of Public Health, Doctor of Public Health and Doctor of Philosophy. The programs range in duration from 2 to 10 years, place special emphasis on urban and global health issues, and have curricula that focus on elements of life and biological sciences, social sciences, and the humanities, as well as epidemiology, health equity, program evaluation and research. The overarching goal of the programs is to provide students with interdisciplinary understanding of public health, using a broad spectrum of approaches and course work (13). Students enrolled in these programs acquire knowledge and skills needed for graduate school, and careers in a wide range of public health and interdisciplinary professions (13).

### Sampling and Data Collection

We conducted a cross-sectional study of students enrolled in GSU SPH programs to obtain information on student academic needs due to COVID-19 and how distance learning was affecting their academic work. Given the fact that not all GSU SPH students and faculty are familiar with distance learning and that some students do not have ready access to Wi-Fi, and are essential workers, the purpose of the study was to (i) identify the challenges GSU SPH students were facing with their academic work as a result of COVID-19; (ii) inform GSU SPH leadership and faculty about the challenges so they can take appropriate steps to address them; and to (iii) identify the positive aspects of the on-line transition as a result of COVID-19.

Students were informed about the study and its purpose through faculty announcements and information posted on GSU SPH and faculty learning management system (iCollege) pages. Students who volunteered to participate in the study completed a one-time 6-minute online survey. Students gave their consent to participate in the study by completing the survey. No personally identifiable information was collected. Quantitative and qualitative data were collected on a daily basis using Qualtrics. All students in the GSU SPH were given an opportunity to participate in the study. The study was exempt from the Georgia State University IRB review process because, it was within the eight exempt categories allowed by the institution and because the study met GSU's ethical standards.

### Variables and Measurement

The study questionnaire comprised 22 multiple choice, closed and open-ended, and Likert Scale questions (ranging from “strongly disagree” to “strongly agree”), and focused on eight domains—demographics, technology and connectivity, online teaching preference (synchronous/asynchronous), academic workload, faculty activities, motivation to learn, behavioral and economic factors, and recommendations. The online teaching preference variable focused on student online teaching preference (synchronous/asynchronous), and the academic load variable focused on whether student academic workload had increased due to the transitioning of all their courses online and the type of assessments they were required to complete. The faculty activities variable focused on faculty adjustment of course syllabi, communication of online class expectations, level of class engagement, faculty accessibility to students and availability of recorded lectures. The motivation variable focused on student ability to complete their assignments on time, remember to login to take quizzes, to communicate with other classmates on class assignments and ability to set time aside to do their schoolwork. The economics and behavioral variable focused on student concerns about they or their family members getting COVID-19, the effects of COVID-19 on their jobs (if they were employed), their financial security and overall effect of the pandemic on their daily life. The recommendation variable gave students the option to provide feedback on positive outcomes of the distance learning and what GSU SPH faculty can do to improve upon their online experience.

### Analysis

Data collected in Qualtrics were cleaned and exported to SPSS for analysis. Univariate, bivariate and multivariate analysis were conducted for quantitative data. Missing quantitative data were excluded from calculations. The IBM SPSS statistical software version 26 was used for the data analysis. Qualitative data were manually extracted from the SPSS database and themes were established using a thematic framework. Analyzed qualitative data are presented in verbatim quotations (in italics) to convey exactly what students said in explanation to certain questions.

## Results

### Univariate Statistics

#### Demographic and Academic Status

Overall, 184 of the 792 (23%) students enrolled in GSU SPH programs invited to participate in the study, completed the survey. Of this number, 35.3% were graduate students (Master of Public Health, Doctor of Philosophy and Doctor of Public Health), and 64.6% were Undergraduate students. Majority of the students (82.6%) self-identified as female. Per employment status, 28.8% of students worked full time, 37.5% part-time and 33.7 were not working - traditional students (Table 1).

**Table 1:** Descriptive characteristics of participants by domains.

**Variables**	**Sample Size, *n***	**Percentage, %**
**1. DEMOGRAPHICS**		
**Academic Status**		
Freshman	10	5.4
Sophomore	7	3.8
Junior	51	27.7
Senior	51	27.7
Graduates	65	35.3
Total	184	100.0
**Gender**		
Male	31	16.8
Female	152	82.6
Other	1	0.5
Total	184	100.0
**Employment Status**		
Full time	53	28.8
Part-time	69	37.5
Not currently working	62	33.7
Total	184	100.0
**2. TECHNOLOGY AND CONNECTIVITY**		
**Device used at home for classwork (Multiple)**		
Desktop PC	20	10.9
Laptop/chrome book	184	100.0
iPad/tablet/kindle	23	12.5
Smart phone	87	47.3
**Internet access at home**		
Broadband	163	88.6
DSL	3	1.6
Cellular service	17	9.2
No Internet access	1	0.5
Total	184	100.0
**3. ONLINE TEACHING PREFERENCE**		
Synchronous	55	30.1
Asynchronous	128	69.9
Total	183	100.0
**Able to access the GSU Internet hotspot**		
Yes	30	17.3
No	143	82.7
Total	173	100.0
**Access to internet for schoolwork at home**		
Limited access (1–2 h)	5	2.7
Medium access (3–4 h)	13	7.1
Unlimited access (4 h or more)	166	90.2
Total	184	100.0
**Success logging online for classes**		
Not at all successful (Never)	1	0.5
Not very successful (5 out of 10 sessions)	9	4.9
Somewhat successful (8 out of 10 sessions)	62	33.7
Very successful (every time)	112	60.9
Total	184	100.0
**4. ONLINE TEACHING PREFERENCE**		
Synchronous	55	30.1
Asynchronous	128	69.9
Total	183	100.0
**5. ACADEMIC WORKLOAD**		
**Transitioning online increased academic work**		
Yes	118	64.5
No	65	35.5
Total	183	100.0
**Additional work that has increased**		
Journaling	2	1.1
Assignments	57	31.8
Reflection papers	13	7.3
Monitoring quizzes	4	2.2
Discussion posts	46	25.7
Other	13	7.3
None	44	24.6
Total	179	100.0

#### Student Technology and Connectivity

When it came to access to technology at home for class work, students indicated that they had access to multiple devices. As shown in Table 1, all students (100%) had a laptop or Chromebook, 47.3% also had a smart phone, 12.5% also had an iPad, tablet or Kindle, and 10.9% also had a personal desktop computer. Regarding internet access at home, 88.6% of students had access to broadband service through a cable company's hotspot and 11% had access through their cellular phones. Only 1 student did not have internet access at home. Although GSU made available internet hotspot services to students to use at home, 82.7% of students were unable to access or use it. Of the students who had access to internet services at home, 90.2% had access for 4 h or more a day. Although 33.7 and 4.9% of students were either unsuccessful at all or somewhat successful respectively, in logging in to participate in online classes, over 60.9% were able to successfully login. Most students indicated a preference for the asynchronous teaching style (69.9%). Some of the reasons (*n* = 107) they provided included not having to deal with technical issues (seven responses), flexibility of class schedules (100 responses), and the fact that they could learn at their own pace. For example, they said:

“*My wifi access isn't reliable. I use xfinitywifi hotspots and sometimes it doesn't work*.
Having recorded lectures available at my convenience ensures I don't miss out on anything”
“*I like pre-recorded lectures so I can listen to them more than once. I can pause and take notes or look something up if I am confused. There are fewer interruptions and I can go at my own pace and watch the videos on my own time”*
“*It allows me to schedule other things around my life that may come up”*.

#### Student Online Teaching Style Preference

Students who preferred the synchronous style of teaching (30.1%) also provided reasons (*n* = 41 responses) that ranged from their ability to interact with faculty and classmates (21 responses), the reduced likelihood of them skipping classes (two responses), to the fact that synchronous classes helps to keep them up to date with lectures, provides them with structure, motivates, and holds them accountable (*n* = 18 responses) for their school work. For example, they indicated that:

“*I am a better learning if I can actually communicate with my instructor during the presentation. That way if concern arises about the subject, I am able to communicate that with my instructor at that time instead of having to wait and hoping they can get to my question or understand what I'm saying at that moment”*
“*Being in a class setting with the professor and other students allows a certain level of interaction and learning that you do not get in asynchronous learning”*
“*I feel motivated to do the work with everyone else. It s more structured, whereas with asynchronous giving myself lectures to be watched at my convenience would allow me to slack off much more.”*

In providing reasons for the preference of a teaching style, some students (*n* = 6) thought both styles worked.

#### Student Academic Workload

Most students (64.5%) indicated that the transition to purely online classes increased their academic workload. The increase in workload was primarily in the form of new assignments that were added including journaling, written assignments, reflection papers, monitoring quizzes, and discussion posts, with written assignments (31.8%) and discussion posts (25.7%) being the primary forms of assessments assigned. For other students (35.5%) there was no increase in course workload (Table 1).

#### Faculty Activities

Regarding faculty activities, 53.9% of students stated that GSU SPH faculty provided an adjusted course syllabus prior to the implementation of online classes for the remainder of the spring 2020 semester, while 3.3% of students reported otherwise. About 40% of students indicated that faculty communicated to them what to expect with the online classes, however 3.4% said otherwise. Per the students who participated in the study, 24.3% of faculty conducted their online classes in an engaging way, 31.8% were accessible for office hours during the transition to online classes, and 48% of faculty posted their recorded lectures on iCollege (Table 1).

#### Student Motivation to Learn

In spite of the challenges associated with the abrupt changes to the spring 2020 semester, more than half (53.6%) of the respondents reported that they were able to stay motivated and complete their assignments on time. Only a small percentage of students (3.4%) reported difficulty with staying motivated to learn. A little over 50% of students surveyed remembered to log in to take scheduled course quizzes online and 47.5% were able to communicate with their colleagues on group course assignments. Less than half (45.1%) of students said they were able to set time aside to focus on and do their schoolwork.

#### Behavioral and Economic Impact on Students

As shown in Table 1, <10% of students said they were concerned about becoming infected with the virus, while 24.7 and 31.3% said they were moderately or extremely concerned, respectively, about contracting the virus. However, the majority of the students (90.1%) were concerned about the effects of COVID-19 on the health of close family members and friends. COVID-19 has had a financial impact on students. 13.4% of the students reported that due to COVID-19, they had lost their jobs, 14% reported being furloughed, and 19.9% said they were working at places where business had slowed down. One area in which an international student indicated they had been financially affected by COVID-19 was the global situation with exchange rates. The student said, “*As an International student, exchange rate has affected my financial sustainability.”* Another area a local student mentioned was the loss of income from rental property. Almost 41% of students indicated that they had not been financially impacted by COVID-19.

For the GSU SPH students who work, 36% experienced a change in their work hours, 16.9% maintained the same hours but were given different schedules, 17.4% had to work longer hours, and 29.8% experienced no change at all. Comments related to work changes or responsibilities included:

“*l am an essential worker so I'm overworked right now”*
“*Because so many people quit at my job, I now work over time. I work 6 days a week. 3 of those days are 11 hour shifts”*
“*I am fortunate enough to continue working from home.”*

Due to COVID-19, the caretaking responsibilities of some students changed−19.4% had to care for a child as childcare was unavailable and 7% had to care for a sick loved one. Generally, 36.8% of students stated that their daily lives had been affected by the pandemic, 31.9% said they had been moderately affected, and 2.2% said they had not been impacted at all.

#### Student Reports of Positive Outcomes of Distance Learning Experience

While the transition to distance learning was abrupt and unnerving to many students, they reported some positive outcomes. Not having to commute to school and subsequently saving money was reported by more than a third of students (*n* = 144). They said:

“*I do not have to commute to campus, which would take at least 2 hours out of my day everyday”*
“*I don't have to spend an hour and $61 to commute to Atlanta campus via MARTA*”
“*I don't have to commute and pay for gas.”*

Other positive outcomes listed included the fact that students had more time to work on assignments and to be with family and friends (21 responses) - “*Because courses have switched to online I now have more time to complete assignments and make sure the assignments submitted are done well.”* Some students learned to manage their time (three responses), others had faculty support (19 responses)*- “I think that some teachers have really shown flexibility and a strong desire to continue to help our class connect and keep things as normal as possible,”* and a few had flexibility and thus, could manage course schedules at their own pace (27 responses). Only a small percentage of students reported that there were no positive outcomes (10 responses).

#### Student Recommendations

In response to what GSU SPH leadership and faculty can do to make the distance-learning experience a better one, students provided 141 qualitative responses, which have been clustered into eight broad recommendations; (i) improve faculty accessibility, communication, teaching (more engaging), and care for students during this time (41 responses), (ii) acknowledge the situation, understand students, and be lenient with grading (30 responses), (iii) reduce course assessments and workload (20 responses), (iv) nothing needs to be done, good job (18 responses), (v)work on technology, provide students with access to devices, internet and academic software (11 responses), (vi) train and provide faculty with resources (seven responses), (vii) conduct graduation ceremony (three responses) and (viii) reduce tuition fees (two responses).

### Bivariate and Multivariate Analysis

We conducted a linear regression analysis to examine the relationship between behavioral and economic factors, faculty activities, academic workload, technology and connectivity (Independent variables), and motivation to learn (dependent variable). These relationships are presented in Table 2. At a significance level of alpha = 0.05 of all the motivation to learn variables, (“I complete my assignments on time, “I remember to log in to take quizzes,” I am able to communicate with class mates on group assignments,” I am able to set time aside to do my school work”) “completion of assignments on time” had a significant bivariate relationship with the COVID-19 behavioral variable “how worried are you about getting COVID-19?”; beta = 0.184, *p*-value = 0.014. Thus, “how worried are you about getting COVID-19?” was chosen as the main independent variable in our multivariate model, and” completion of assignments on time as our main dependent variable.”

**Table 2:** Bivariate analysis of behavioral and economic factors, faculty activities, academic workload, and technology and connectivity vs. motivation to learn.

**Variables**	**Regression Coefficient**	***P*-value**	**95% Confidence Interval**
**Behavioral and Economic Factors**			
How concerned are you about getting COVID-19?	0.184	0.014	0.035–0.302
Do you have concerns about the health of family or close friends because of COVID-19?	−0.002	0.979	−0.561 to 0.547
**Job Related Factors**			
How is COVID-19 affecting your financial security?			
Lost job or furloughed or business has slowed down	0.007	0.931	−0.324 to 0.354
As a result of the COVID-19 outbreak, have your work hours changed?	−0.008	0.912	−0.160 to 0.143
**Care-Taking Responsibilities**			
Caring for child while school or childcare is unavailable	0.202	0.007	0.159 to 0.982
Caring for a sick loved one	0.024	0.750	−0.538 to 0.745
**Overall**			
COVID-19 affecting day to day life overall	−0.092	0.221	−0.277 to 0.064
**Faculty Activities**			
Faculty provided an adjusted online syllabus prior to the beginning of online classes	0.314	<0.001	0.192–0.506
Faculty communicated what to expect with regards to online classes.	0.368	<0.001	0.242–0.534
Faculty conduct(ed) online classes in a way that is engaging	0.280	<0.001	0.127–0.390
Faculty are just as accessible for office hours since the transition to online classes	0.351	<0.001	0.188–0.435
Faculty make recorded class lectures available at iCollege	0.263	<0.001	0.111–0.381
**Academic Workload**			
Transitioning to online classes increased academic workload	0.122	0.105	−0.020 to 0.209
**What additional work has increased your academic workload?**			
Journaling	−0.030	0.694	−1.934 to 1.291
Assignments	−0.054	0.548	−0.550 to 0.293
Reflection paper	−0.067	0.411	−1.091 to 0.448
Monitoring quizzes	−0.010	0.903	−1.231 to 1.088
Discussion posts	−0.109	0.220	−0.724 to 0.168
**Technology and Connectivity**			
How do you access the Internet at home?			
Broadband (through a cable company's hotspot)	0.618	0.052	−0.018 to 4.421
DSL (through my phone)	0.191	0.200	−0.888 to 4.222
Cellular service	0.540	0.068	−0.156 to 4.406
If needed, are you able to access the GSU Internet hotspot?	−0.149	0.054	−0.912 to 0.007
How much access do you have to the internet to do your schoolwork at home?	0.189	0.011	0.127 to 0.976
Overall, how successful have you been in logging on to participate in classes online?	0.380	<0.001	0.441 to 0.939
**Which online teaching style do you prefer?**			
Synchronous learning (live lectures given at a designated time and day) vs. Asynchronous	−0.008	0.917	−0.127 to 0.114

Statistically significant relationships were also found between student motivation to learn and faculty provision of adjusted online class syllabi prior to the commencement of online classes (*p* = 0.001), faculty communication of what to expect with regards to online classes (*p* = 0.001), faculty facilitation of online classes in an engaging way (*p* = 0.001), faculty accessibility for office hours (*p* = 0.001), and faculty posting of recorded class lectures on the schools learning management system (*p* = 0.001). We did not find statistically significant relationships between student motivation to learn and academic workload (*p* = 0.105), technology and connectivity (*p* = 0.052), and teaching style (synchronous or asynchronous) (*p* = 0.917).

With the multivariate linear regression analysis, after controlling for all variables in the model (Table 3), we found that the less concern students expressed about getting COVID−19, the more likely they were to complete their assignments on time (beta = 0.155, *p*-value = 0.032). We also found that the more faculty communicated what to expect for online classes, the more likely students were to complete their assignments on time (beta = 0.362, *p*-value < 0.001). The more successes students reported for logging online to participate in class, the more likely they were to complete their assignments on time (beta = 0.247, *p*-value < 0.002). There was no statistically significant relationship between transitioning of classes online and motivation to complete assignments on time (*p* = 0.454). There was also no statistically significant relationship between academic status, gender, employment status and motivation to complete assignments on time.

**Table 3:** Multivariate analysis of motivation to complete assignments on time and other COVID-19 associated factors (behavioral/economic, faculty activities, academic workload and technology and connectivity).

**Variables**	**Regression Coefficient**	***P*-value**	**95% Confidence Interval**
**Behavioral and Economic Factors**			
1. How concerned are you about getting COVID-19?	0.155	0.032	0.012 to 0.264
2. Caring for child while school or childcare is unavailable	0.132	0.073	−0.034 to 0.750
3. Lost job or furloughed or business has slowed down vs. working or not applicable	−0.041	0.612	−0.201 to 0.119
**Faculty Activities**			
4. Faculty communicated what to expect with regards to online classes.	0.362	<0.001	0.231 to 0.536
**Academic Workload**			
5. Transitioning to online classes increased academic workload	0.054	0.454	−0.067 to 0.149
**Technology and Connectivity**			
6. Broadband (through a cable company's hotspot)	0.153	0.330	−0.532 to 0.536
7. Cellular service vs. none	0.104	0.496	−0.737 to 1.515
8. Overall, how successful have you been in logging on to participate in classes online?	0.247	0.002	0.168 to 0.698
**Demographics**			
9. Male vs. Female	0.028	0.684	−0.318 to 0.483
10. Sophomore vs. Freshman	−0.035	0.689	−1.222 to 0.810
11. Junior vs. Freshman	0.006	0.969	−0.674 to 0.701
12. Senior vs. Freshman	−0.017	0.907	−0.717 to 0. 637
13. Masters vs. Freshman	−0.019	0.885	−0.763 to 0.658
14. PHD vs. Freshman	−0.060	0.545	−1.179 to 0. 625
15. DrPH vs. Freshman	−0.057	0.623	−1.033 to 0. 620
16. Employment status (Full time vs. not currently working)	−0.042	0.658	−0.545 to 0.345
17. Employment status (Part time vs. not currently working)	−0.154	0.072	−0.716 to 0. 031

## Discussion

The study was conducted to identify challenges GSU SPH students experienced as a result of the transitioning of all courses online in response to COVID-19, what faculty need to do to address the challenges that students faced, and also to identify the positive efforts that faculty made during the transition. Findings from the study provide information on the challenges and potential benefits GSU SPH students experienced as a result of the transition of all courses to an online format owing to COVID-19 for the rest of the fall 2020 semester. The results also provide information on the strategies employed by GSU SPH faculty that positively impacted student motivation during the pandemic, those that need to be improved upon, and those which can be replicated going forward.

### Student Technology and Connectivity

Prior to the study, GSU SPH faculty were concerned that students would have difficulty accessing and participating in courses online due to challenges associated with access to technology off campus. There was also a concern that this challenge along with other factors would affect student motivation to learn, academic performance, and success in classes during the spring 2020 semester. Faculty concerns are well documented in the literature. A study conducted by Liu et al. confirmed that lack of access to technology by college students has the tendency to negatively impact their learning outcomes (14). Study results however showed the contrary, only one of the 184 students surveyed did not have access to a technological device. Nonetheless, access to a technological device does not guarantee access to internet services.

Although GSU SPH provided students with access to the Schools hotspot, study results found that many could not use those hotspots to access the internet. Students who were unable to access internet services had to find alternative means. Given that distance learning will continue for the foreseeable future, it is imperative for GSU SPH leadership to further examine this matter and seek viable solutions to assisting students with gaining reliable internet access. This is a matter for the success of our students, which is inexplicably tied to the future of higher education overall.

### Student Online Teaching Style Preference

The majority of students indicated a preference for the asynchronous approach to online teaching. Their choice was primarily based on the fact that it gave them the ability to learn at their own pace, and to do course work when they were ready. Additionally, having access to pre-recorded course lectures and other resources was convenient and enhanced their ability to manage their schedule from wherever and at whatever time. This finding is consistent with what Trach noted and what GSU SPH faculty found based on evidence from a small unpublished student survey that was conducted when the GSU the Bachelor of Science in Public Health (BSPH) program was initially established in 2017. When asked to rank their preferred course offering modality, the majority (46%) of pre-public health students, opted for the 100% online (asynchronous) offering. According to Trach, asynchronous learning gives students the ability to access course information, demonstrate what they have learned, and to communicate with classmates and instructors on their own time without having to be in the same classroom or same time zone. To Trach, asynchronous learning not only provides flexibility for non-traditional students, but also accommodates different learning styles, as students can choose the order they wish to cover material and how much time they want to dedicate to delving into a particular class (15). On the other hand, some students said the synchronous approach was the better option in that, it gave them the opportunity to participate in live streamed lectures, allowed for high faculty-student interaction, and the receipt of immediate feedback on course material. It also gave them some structure and caused them not to be lazy with their schoolwork. In their study, Lobel et al. found that one frequently mentioned advantage of the synchronous learning method, was the spontaneous and dynamic nature of interactions that the asynchronous method does not support (16). Other students preferred a blend of the synchronous and asynchronous style of teaching.

### Student Academic Workload

Although students indicated that their academic workload significantly increased as a result of the transition to distance learning, it did not affect their motivation to learn or to complete their assignments on time. This shows that irrespective of the challenges, students were determined to complete the semester with good grades. It also suggests that the best way forward with distance learning will be to adjust expectations, and to reduce assessments that overly test online participation and content assimilation. The best way forward will also require faculty to check on students from time to time and to talk with them about their academic challenges to strike the right balance and not overburden students. Some students indicated that faculty engagement in such activities will be very helpful.

### Faculty Activities

In preparation for the transition to a full distance learning format, GSU SPH faculty engaged in a series of preparatory activities to make the transition a smooth one. Results from the study show that the attention that was given to preparing faculty for meeting student needs in the online format was communicating course expectations to students, making available recorded class lectures and being accessible for office hours. Investigations from Dziuban et al. demonstrate that students show a high probability of assigning an excellent overall rating for faculty who in their view, facilitate learning, effectively communicate course materials and information, organize courses effectively, assesses student progress accurately, show interest in students' learning, and show respect for their students (17).

### Student Motivation to Learn

Over 50% of students surveyed indicated that they were motivated to learn regardless of the learning environment. Thus, it was not surprising that they completed their assignments and turned them in on time and also remembered to log in to take quizzes. Some aspects of student motivation can be attributed to their access to faculty during the semester, their ability to access course materials and recorded lectures asynchronously, and the flexibility of schedules. Schunk et al. define motivation as the process whereby goal-directed activity is instigated and sustained (18). They indicate that one's motivation can influence what they learn, how they learn, and when they choose to learn (19). According to available literature, motivated learners are more likely to engage in challenging activities, be actively engaged, adopt an approach to learning, and exhibit enhanced performance and persistence (18) even under challenging circumstances.

### Behavioral and Economic Impact on Students

Not as many students were worried about contracting COVID-19 (24.7%). Financially, the majority of students (66.1%) had their economic activities disrupted by the pandemic. As was predicted by economists, the implementation of mitigation efforts around the world to counteract the tightening grip of COVID-19 will accelerate job losses and create new schedules. This is exactly what happened to GSU SPH students who work (20). Consistent with what GSU SPH faculty presumed, a little under 37% of students said that overall, their daily lives had been affected by the pandemic a lot. With unimaginable national response measures in place to contain the pandemic, families have been separated, public spaces have been closed, and economic activity has drastically slowed down, creating a new normal, and rearranging the lives of GSU SP|H students to fit into the shifting land scape (21). GSU SPH international students who have no home in the US except campus, had to cope not only with the closure, but also with the fear that inflation arising from global economic inactivity, could potentially affect their scholarships.

### Student Positive Outcomes of Distance Learning

As students were still processing the shock of campus closure and adjusting to the new world of predominantly online classes, the general consensus was that they would miss out on face-to-face interaction with faculty and their peers. This is because, in any given semester, students do not usually enroll in only online courses- they usually go for a combination. After analyzing the data collected, it became apparent that many students were okay with not having to be in the physical presence of faculty - in so far as faculty communicated course expectations, were available, and made course materials and assessments available. Less commuting, saving on gas, having more time to do assignments, time management, and spending time with family were some of the positive outcomes proffered for the closure of campus. Additional positive outcomes stated included having more time to rest, increased communication with faculty, and obtaining leniency with assignment submission dates. The responses associated with time management were unforeseen.

### Student Recommendations

Students provided a number of recommendations in response to the question related to what GSU SPH leadership and faculty can do to improve the distance learning experience. After analysis of the qualitative statements made by the students regarding recommendations, the following six themes emerged; (i) Need for reliable technology; (ii) more flexibility in assessments and grading; (iii) improve faculty access and response times to student correspondence; (iv) adjustment to student course workload; (v) faculty preparation for online teaching; and (vi) facilitate engaging content for synchronous classes. Practices that did not enhance their experience will be addressed and those that were positive will be documented and replicated in the future. Research shows that educational experiences that are active, engaging, and student-owned lead to deeper learning.

## Study Limitations

Limitations to this study include the fact that <25% of the GSU SPH student population participated in the study. While the data is supportive of the efforts that SPH faculty and leadership have exerted in facilitating effective, relevant pedagogy during this crisis, it would have been preferable to have at least one-third of the student body respond. Additionally, the study is limited in that, it was conducted in unprecedented times, where there was a high likelihood that students' emotional levels could have affected their perceptions of the impact of the online transition.

## Conclusion

Despite the unprecedented events that led to the need for GSU SPH students to conclude the spring 2020 semester via distance learning approaches, this study found that students were still motivated to learn and to complete their assessments and assignments on time. Considering that the abrupt and unforeseen changes also had an impact on faculty teaching, motivation and preparation, student recommendations for SPH leadership and faculty to take certain measures to make their distance learning better going forward, were documented and will provide evidence for changes in the future. This study is specific to student outcomes only at GSU SPH, however, some of the recommendations provided by students may be pertinent to other institutions of higher learning.

## Data Availability Statement

All datasets generated for this study are included in the article/supplementary material.

## Ethics Statement

The studies involving human participants were reviewed and approved by Georgia State University Institutional Review Board. The patients/participants provided their written informed consent to participate in this study.

## Author Contributions

EA-M wrote the draft manuscript, did the qualitative data analysis, finalized, and edited the manuscript. KR-W wrote a portion of the draft manuscript and edited the manuscript. BY performed the statistical analysis. SS-B edited the manuscript. All authors contributed to the article and approved the submitted version.

## Conflict of Interest

The authors declare that the research was conducted in the absence of any commercial or financial relationships that could be construed as a potential conflict of interest.
